# The need to be unique and the innovative behavior: The moderating role of supervisor support

**DOI:** 10.3389/fpsyg.2022.979909

**Published:** 2022-09-01

**Authors:** Mustafa Bekmezci, Wasim Ul Rehman, Muzammil Khurshid, Kemal Eroğluer, Inci Yilmazli Trout

**Affiliations:** ^1^Department of Defense Management, National Defense University, Turkish Military Academy, Istanbul, Turkey; ^2^Department of Business Administration, Faculty of Business, Economics and Administrative Sciences, University of the Punjab, Gujranwala, Pakistan; ^3^Department of Banking and Finance, Faculty of Commerce, University of the Punjab, Gujranwala, Pakistan; ^4^Department of Business Administration, National Defense University Turkish Military Academy, Ankara, Turkey; ^5^Dreeben School of Education, University of the Incarnate Word, San Antonio, TX, United States

**Keywords:** supervisor support, need for uniqueness, innovative behavior, Turkey, workplace

## Abstract

The purpose of this study is to examine the moderating effect of supervisor support on the relationship between the need to be unique and the innovative behavior. People not only strive to belong to a group but also want to be unique from others and feel exceptional. Individuals’ innovative behavior is one of the things that makes them feel different from other people. Because developing a new idea, supporting this idea, putting this idea into practice, and the positive achievements of this idea distinguish people who exhibit innovative behavior from others. It depends on the behavior of supervisors whether people who break away from typical practices and procedures to feel unique and special continue to act in innovative ways. In this context, it is vitally essential for supervisors to support people who display innovative behavior. The research was conducted on employees working in the education sector in Mersin. We employed the confirmatory factor analysis (CFA) to examine the fitness of the model and moderation was tested. As hypothesized, the need to be unique had a significant and positive effect on innovative behavior. This finding is consistent with existing literature and thus advance knowledge on need to be unique and innovative behavior, particular in education sector. Nonetheless, it has been determined that supervisor support doesn’t have a moderator role on the relationship between the need to be unique and innovative behavior. Although there are some researches in the literature on consumer experiences about the need to be unique and innovative behavior, but literature on education section is sparse and still long way to go to evaluate its’ reflections on the workplace.

## Introduction

Human beings are social animals. Throughout the evolution of the human species, belonging has ensured human survival and has been one of the most basic human needs. To be a group member, man has always tried to fit in, to demonstrate characteristics that conform to the group and be accepted and liked by the group (Festinger, 1950). As a matter of fact, there have been numerous researches on this topic (Schumpe and Erb, 2015, p. 1). However, people not only strive to belong to a group but also want to feel different and unique from others. People’s drive is defined as the need for uniqueness (Schumpe and Erb, 2015, p. 1). People examine one another, compare themselves to others, notice similarities, and make an effort to stand out from the crowd. According to an analysis of 2,000 randomly selected advertising from the best-selling tabloids from 1900 to 1980, the concept of being different from others/originality/uniqueness is employed as the central theme in 10% of the advertisements and as a subsidiary theme in 23% (Lynn and Harris, 1997, p. 1).

It can be stated that innovation is one of the elements that make people feel unique from others because individuals who innovate by using ideas that are unique from those of others will differentiate themselves from others. Additionally, innovation gives the innovator independence, possibilities for learning and self-development, enhances their abilities, decreases their workload, and improves the organization’s performance (Özbey and Başdaş, 2018, p. 3). These benefits of innovation will enable the emergence of new products and services, the reduction of costs, the production of alternative solutions, effective and efficient business processes, and the appropriate use of limited resources. Therefore, by exhibiting innovative behavior, people will both differentiate themselves from the crowd and contribute to the organization. However, this may require the person to break the rules from time to time, to defend their own thoughts and ideas, and to remain unresponsive to opposing thoughts.

On the other hand, researchers suggest that supervisor support is vital to motivate employees for showing innovative behavior (Anderson et al., 2014). If supervisors demonstrate that they care about their employees’ security, well-being and value their assistance, this will consequently enhance their innovative behaviors (Anderson et al., 2014). Therefore, even if employees occasionally break the law, supervisors should encourage their innovative behavior. Practices such as employees’ strict adherence to the rules, non-participation in decisions, obtaining the approval of the supervisor in all matters, and not supporting differences are actions that prevent creativity, which is the source of innovation. In this context, the quality of the relationship between the employee and the supervisor and the supervisor’s creating a suitable environment for the employees to act innovatively are extremely important for the employees to express themselves and feel special. Managing innovative behavior requires a focus on both idea implementation by creating supportive climates and conditions (e.g., favorable structures, policies, and resources) for both stages (Carnevale et al., 2017, p. 523).

In this study, the effect of supervisor support on exhibiting innovative behavior of teachers who need to be unique from others was investigated. Every person has a different learning style and method, everyone’s intelligence type and intelligence level is different. For this reason, teachers’ teaching of any subject in different ways and using new methods by which students can understand the subject will increase the learning level of the students. In addition, the fact that the students are more successful than the students in other classes thanks to the teacher’s innovative behaviors will differentiate the teacher from his/her colleagues and will make him/her feel unique and special. The fact that supervisor support these practices that increase the learning level of students will increase the motivation of teachers. Because the continuity of innovative behavior depends on the supervisor’s approach.

## Literature review and development of hypotheses

### The need to be unique

The need to be unique involves “making a conscious attempt to stand out from the crowd.” (Snyder and Fromkin, 1977, p. 518). The theory of being different, uniqueness, and authenticity asserts that interpersonal similarity has emotional, cognitive, and behavioral consequences (Schumpe and Erb, 2015, p. 2; Lynn and Snyder, 2002, p. 395). According to the idea, a person’s likeness to other people is too low or too excessive, causing discomfort and adverse emotional reactions. Although the individual seeks more differences to demonstrate her/his identity (Okamoto, 1983, p. 69), a moderate degree of similarity elicits the most pleasant emotional reaction (Lynn and Harris, 1997, p. 2; Schumpe and Erb, 2015, p. 2). Fromkin (1972) used a lifestyle survey to students to investigate this theory. One set of students was told their answers were highly similar to those of other students, another group was told their responses were different or pretty different, and yet another group was told their answers were moderately similar to those of other students; the students were then asked to rate their own mood after receiving this information. Confirming the theory, respondents who were reasonably similar to other participants had a more positive mood than those who were very similar to or entirely dissimilar from other participants.

The need to be unique is a psychological state related to how similar an individual feels to other people and inspires compensatory measures to establish a sense of being unique (Imhoff and Erb, 2009). As a result, according to the theory of difference, if an individual is similar to others, he/she feels threatened and responds in a way that distinguishes himself/herself from others whenever he/she detects this threat. In order to preserve their distinctiveness/authenticity, people may exhibit behaviors such as ignoring information that threatens its originality, remembering information that supports its uniqueness, focusing on this information, or increasing the level of distinctiveness of their attitudes and behaviors (Lynn and Snyder, 2002, p. 396).

People feel unique from others may also be due to individual differences, and some people may want to be different from others. This predisposition has been discovered to be a significant predictor of unique behavior (Lalot et al., 2017, p. 1). In fact, it has been shown that people with a strong desire to stand out are more likely to adopt new items and brands (Latter et al., 2010, p. 2).

The scales established by Snyder and Fromkin (1977) for the Need for Uniqueness (NfU) and Lynn and Harris (1997) for the Self-Attributed Need for Uniqueness (SANU) are primarily utilized in the field of psychology. While NfU emphasizes mostly public and risky differentiation indicators, SANU focuses on personalized and socially acceptable means to achieve a sense of being unique.

Snyder and Fromkin (1977) created a scale with three dimensions: the desire to stand out, the individual’s disrespect for other people’s opinions, actions, and reactions, the individual’s refusal to follow the rules constantly, and the willingness to defend one’s convictions in public. Because of the significant correlation between items, NfU was commonly utilized as a one-dimensional scale despite its multidimensional nature (Lalot et al., 2017, p. 2; Schumpe et al., 2016,p. 232). NfU has also been studied in social psychology and related fields such as consumer behavior, societal influences, and cultural and individual differences (Schumpe et al., 2016). Studies have shown a positive relationship between NfU and personality traits such as risk-taking and novelty-seeking (Lalot et al., 2017, p. 2). Furthermore, a positive correlation was found between NfU’s personality traits of extraversion (Kagan and Esquerra, 1984; Dollinger, 2003) and openness to experience (Dollinger, 2003; Şimşek and Yalinçetin, 2010). This suggests that people with a strong desire to stand out are more active, social, extroverted, and open to new experiences (Schumpe and Erb, 2015, p. 5). The personality trait neuroticism was found to have a negative connection with NfU (Dollinger, 2003). This suggests that people with a solid need to be unique are more emotionally stable and satisfied with their lives than people with a low need to be unique (Schumpe and Erb, 2015, p. 5). Furthermore, it was discovered that societies with collectivist cultures scored lower on the urge to be different from societies with individualistic cultures (e.g., Burns and Brady, 1992; Tafarodi et al., 2004).

NfU assesses people’s public attitudes and behaviors, giving undue weight to indicators that could be regarded as socially dangerous (Lynn and Harris, 1997, p. 10). In this context, being different is demonstrated by ignoring others’ reactions, disobeying regulations, and refusing to participate publicly with others. However, such behaviors may enrage others, and those who engage in them may be isolated from society. Also, NfU admits that when an individual is threatened, they relinquish their tendency to be different from others (Schumpe et al., 2016, p. 231). As such, it can be said that NfU adopts an indirect and contextual approach (Lalot et al., 2017, p. 2). Even if a person’s desire to be unique is limited to socially acceptable behaviors, that person’s originality cannot be reliably quantified. People can also keep their uniqueness by engaging in more specific or socially acceptable behaviors. Lynn and Harris (1997) criticized NfU on these grounds. They developed the Self-Attributed Need for Uniqueness (SANU), a four-item, a one-dimensional alternative to NfU that individualized the need to be different, the individual’s preference to be different from others, the importance of being different from others, how often one does something to make a difference. How different one is from other people. Lynn and Harris (1997) examined the relationships between the self-perceived need to be unique and some consumer trends, such as consumers’ desire for rare, innovative, and personalized products and their preferences for unusual shopping malls, rather than consumers’ sensitivity to normative influences, and discovered that these relations were mediated by a latent variable that reflected individual differences in the desire to be unique through consumption.

The urge to be unique indicates that everyone has a moderate need or desire to be different from others. However, the state of being different is limited by the need for social acceptance and approval, so people try to be authentic by avoiding behaviors that isolate them from society or are not approved by society (Lynn and Harris, 1997, p. 2). The need to be unique is also a stable personality trait that can vary depending on the situation (Schumpe and Erb, 2015, p. 3). The desire to be optimally unique differs from person to person. It can also determine how distinct and unique a person wishes to feel from others (Schumpe and Erb, 2015, p. 3). Although there has been much research on group identity, consumer products and consumer experiences within the scope of the need to be unique (e.g., Sungur, 2018; e.g., Workman and Kidd, 2000), no research has been found on the reflections of the need to be different in the workplace. Knowing and assessing the employees’ need or want, structuring the working environment based on the results, educating people to meet these demands, enhancing motivation, job and life satisfaction, and organizational commitment can all help.

### Supervisor support

As social beings, people want to be accepted and supported throughout their lives. This stands true for the workplace as well. Employees perceive how much their institutions value their contributions and how much they care about their well-being; Eisenberger et al. (1986) defined this perception as perceived organizational involvement. Studies have shown that organizational support increases participation, performance expectation, and innovative behavior and reduces labour turnover and absenteeism (Yoon and Lim, 1999, p. 924). Organizational involvement is a result of the organisation’s policies and practices. It is the belief that the organization is ready to reward the employee’s effort to satisfy the demand for praise and approval and to demonstrate how highly it appreciates the contributions of its people and cares about their well-being (Eisenberger et al., 1986, p. 501; Rhoades and Eisenberger, 2002, p. 699). In other words, organizational involvement is the perception of employees that the organization values their contribution to the organization and considers their well-being (Yüksel, 2006, p. 8). Employees will feel that the organization supports them as long as they are acknowledged and respected, their needs for relationships are addressed, and their efforts are rewarded (Giray and Şahin, 2012, p. 2).

Social support, which includes assistance from colleagues and supervisors and is handled differently depending on its structure and form, can also be evaluated in the context of organizational involvement. Supervisor support has been evaluated as one of the precursors of organizational involvement (Rhoades and Eisenberger, 2002; Allen et al., 2003). However, it has been determined that the source of organizational involvement and supervisor support is different and that employees can distinguish support from these two sources (Giray and Şahin, 2012, p. 2).

House (1981) mentioned four types of organizational involvement. These are; emotional support (e.g., love and empathy), instrumental support (e.g., goods and services), informational support (e.g., information about the environment) and appraisal support (e.g., information about self-assessment). Based on House’s (1981) definition Bhanthumnavin (2000) defined social support as an interpersonal behavior in which the provider supports the recipient in a given situation and states the support that the provider can give to the recipient as follows: (1) emotional support (e.g., showing empathy and care, validation), (2) informational support (e.g., giving feedback, providing guidance on work-related knowledge and skills, giving advice), (3) material support (e.g., preparing budgets, benefits, resources, and other financial matters related to work). In this context, it is possible to define supervisory support as the emotional and financial support of the employees by their first supervisor (Yoon and Lim, 1999, p. 925) and informing them about the issues they need. Supervisory support is a positive business interaction between supervisor and subordinate and the supervisor’s response to subordinates’ demand for motivation to work more effectively (Bhanthumnavin, 2003, p. 79). This approach ensures the establishment of relations between the supervisor and their subordinates and turns into a partnership that includes support, trust, knowledge sharing, liking, respect and reciprocal influence (Gagnon and Michael, 2004, p. 173).

It has been found that supervisor support improves employee attitude and performance (Nystrom, 1990, p. 298). Eisenberger et al. (1990, p. 57) found that employees’ general perceptions that they are valued and cared about by the organization are positively associated with conscientiousness in carrying out conventional job responsibilities, participation, and innovation. Supervisor support also increases work engagement (Turgut, 2011, p. 161). Gomes et al. (2015) also stated that innovation would manifest itself when individuals feel engaged in their work, and as a result of their research, they found a positive relationship between self-leadership, work engagement and individual innovation. In addition, cynicism and leaving the job are both stated as being strongly influenced by the lack of feedback, job control, social support, and involvement in the decision process (Schaufeli et al., 2002, p. 87). Yoon et al. (1996) stated that organizational involvement and supervisor support are more substantial than all other sources of support and showed that both forms of support are empowerment mechanisms that increase the sense of control, regardless of other work and organizational aspects. It has been established that employee empowerment significantly benefits innovation (Uzunbacak, 2015).

In studies on teachers, it has been determined that supervisor support increases job performance, job satisfaction (Uzun and Özdem, 2017) and emotional commitment (Li et al., 2018). In their longitudinal study on teachers, Burke et al. (1996) also found that lack of supervisor support is among the causes of job stress.

### Innovative behavior

Nowadays, the success of organizations depends on being innovative (McAdam and Keogh, 2004). The degree of innovation within a business organization is also influenced by the degree of innovation among its employees (Şekkeli, 2021, p. 60). In other words, the business organization’s innovation depends on its employees’ innovative behavior (Thurlings et al., 2014, p. 1). Yuan and Woodman (2010) stated that it is vitally important to have an innovative employee in a dynamic environment. For this reason, it is of great importance that the supervision supports innovation, eliminates the factors that prevent innovation, provides the necessary physical environment for innovation and that its processes are appropriate for innovation (Karamehmet, 2012, p. 3). In this context, besides R&D activities, the behavior and perspective of the employee gain importance. Hurt et al. (1977, p. 59) stated that it is a more correct approach to define innovativeness as a personality structure. Innovative behavior is about an employee’s willingness to innovate on the job (Dorenbosch et al., 2005, p. 129), the behavior towards consciously creating, promoting and implementing a new idea (Janssen, 2000, p. 288). A small change resulting from innovative behavior can generate massive revenue for the business organization (Abbas and Raja, 2015, p. 129). For this reason, innovative behavior is considered a strategic activity that brings a competitive advantage to the business organisation, and for this reason, many organisations encourage their employees to be innovative (Turgut and Beğenirbaş, 2013, p. 108). Not only do organizations in highly competitive markets need to innovate, so do nonprofit organizations, such as educational institutes. Likewise, the environment in which schools operate changes rapidly due to more varied student populations, expanding knowledge fields, new responsibilities, and higher social expectations of schools (Thurlings et al., 2014, p. 2). The reason why employees innovate is to increase performance (Yuan and Woodman, 2010, p. 325). As a matter of fact, it is claimed that the developments in innovation improve the performance of the business organization (Damanpour, 1991). Additionally, employees like their occupations more since they can express their creativity at work. Even in professions where processes are tightly defined, innovative behavior can be displayed by employees who enjoy their jobs. For example, in a study of the housekeepers at Disney World, one employee arranged the plush toys in the room as if they were playing different scenes every day, and another employee checked the room by lying on the bed because it was determined that the guest, who had a long day, behaved like this since the employee knew that this was the first thing they would do when they returned to the room (Buckingham, 2022, p. 57). Even such a small amount of autonomy causes employees to love their jobs and increases customer satisfaction. Individual innovative behavior begins with defining the problem and generating new or adopted solutions (Scott and Bruce, 1994, p. 581). Within the scope of this information, innovative behavior can be defined as the development, adoption and application of new perspectives for products and business methods (Yuan and Woodman, 2010, p. 323).

Innovative behavior and creativity are frequently confused. Creativity is about finding new ideas, while innovative behavior is about finding new ideas and applying those ideas (Scott and Bruce, 1994, p. 581). In this regard, producing creative ideas is a component of innovative behavior (Yuan and Woodman, 2010, p. 323). It should also be noted that there are different antecedents of innovative behavior. Among these elements; psychological freedom and safety (e.g., West and Wallace, 1991), self-assurance (e.g., Barron and Harrington, 1981), intrinsic motivation (e.g., Jung, 2001), psychological capital (e.g., Avey et al., 2010), the relationship between the employee and the supervisor (e.g., Janssen and Van Yperen, 2004), leadership philosophies (e.g., Scott and Bruce, 1994), organizational context, (e.g., Oldham and Cummings, 1996), innovation strategy (e.g., van der Panne et al., 2003), and discomfort with the present circumstance (e.g., Yuan and Woodman, 2010) can be discussed.

Innovative behavior can be technological (in products, services, production processes), administrative (changes in activities, social processes, structures) or radical, depending on how it affects existing products or processes (Damanpour, 1991). Therefore, the innovativeness of the employees during the innovation process; for example, it can be traced from idea generation to product development and commercialization of the developed product or adoption of new processes or patterns in the business (Vincent et al., 2002).

There are three main reasons why innovative teacher behavior in schools is needed (Thurlings et al., 2014, p. 2). First, innovative behavior is important to keep up to date with a rapidly changing society. Second, upcoming new technologies and new insights about teaching require innovative behavior. Third, schools should set a good example and act as a starting point for more innovative behavior of our citizens so that society can stay competitive. So, it is important to enhance teacher innovative behavior.

Leadership is the most essential element that reveals creativity (Subramaniam, 2012). Besides supervisor’s support has been shown to be an effective antecedent of employees’ innovation behavior and creativity and organization’s supervisors can positively influence employees’ motivation, satisfaction, and can create a positive atmosphere, which encourages innovative behavior among employees (Darvishmotevali, 2019, p. 37). In addition, Scott and Bruce (1994) discovered that the leader-member exchange theory has an impact on innovative behavior and that innovative behavior is influenced by the climate for innovation, leadership, work groups, and problem-solving style. Yuan and Woodman (2010) stated that supervisor relationship quality would positively affect the innovative behavior results of employees. Sazkaya and Dede (2018) determined that employees need authority to exhibit innovative behavior, and this is possible by removing the traditional barriers between the supervisor and the employees. Similarly, Darvishmotevali (2019) found that supervisor support strengthens the positive impact of decentralization on innovative behavior. Kanter (1983) and Peters and Waterman (1982) established that innovation is more likely to occur when the leadership style is participative and collaborative.

In studies on teachers, Binnewies and Gromer (2012) explored supervisor support related significantly to idea generation and promotion but not to implementation. Noefer et al. (2009), found that supervisor feedback positively affected idea generation. Bourgonjon et al. (2013) demonstrated that a critical mass, meaning if a large group of people in the same organization, or even in society, positively influenced innovative behavior, used ICT, teachers were more inclined to use ICT in their own classes. In other words, if a critical mass was perceived, teachers’ beliefs about the usefulness of ICT were also positively influenced. On the other hand, colleagues can greatly limit innovative behavior by not supporting innovation, for example, when there is an egalitarian teacher culture. However, positive results of innovative behavior on students were found (Ross and Bruce, 2007).

*H1*: The need to be unique significantly and positively affects innovative behavior.

*H2*: The supervisor support has a moderating role between the need to be unique and innovative behavior.

## Methods and instrumentation

In this research, which was conducted to determine the effect of the need to be unique on innovative behavior and the moderating role of managerial support in this effect, firstly, information was given about the sample and the scales used to collect data, and then an analysis of the model was made. In this context, confirmatory factor analysis of each variable was performed, the correlation between the variables was examined, and the moderator effect was tested. Before beginning the study, the data was examined to see if it was normally distributed, if there were any missing values and if there were any extreme values.

In this research, the survey technique was used to collect data. The information was gathered by emailing the survey form to the participants *via* WhatsApp. There are two sections to the survey, each with 27 questions. There are six questions regarding the respondents’ demographic information in the first section of the survey and 21 questions about the need to be unique, supervisor support, and innovative behavior in the second section.

### A sample of the study

The majority of the participants in the study are educators who work in both the public and private sectors in Mersin. A total of 403 people responded, 25 of whom were eliminated from the review and 380 of whom were found to be appropriate for analysis. 41.3% (*n* = 157) of the participants were female, 78.2% (*n* = 297) were married, 6.84% (*n* = 26) were between the ages of 20–30, 33.15% (*n* = 126) were between the ages of 31–40, 40.0% (*n* = 152) were between the ages of 41–50, 17.89% (*n* = 68) were between the ages of 51–60, and 2.10% (*n* = 8) were 61 years old and above. A total of 78.9% (*n* = 300) of respondents work in the public sector, while 21.1% (*n* = 80) work in the private sector. The participants had an average of 18.23 years of work experience.

### The scale of the need to be unique

The Self-Attributed Need for Uniqueness Scale developed by Lynn and Harris (1997) was used to determine the participants’ perceptions of the urge to be different. The Self-Attributed Need for Uniqueness Scale (SANU; Lynn and Harris, 1997) is a 4-item scale which evaluates an individual’s perception of his or her own need for uniqueness. Higher scores are indicative of a higher need for uniqueness. Some of the expressions of the scale are as follows: “I prefer _____ different from other people. [(a) no, (b) slightly, (c) moderately, (d) very, (e) extremely],” “I have _____ a need for uniqueness. [(a) weak, (b) slight, (c) moderate, (d) strong, (e) very strong].” Lynn and Harris (1997) obtained a Cronbach’s alpha coefficient of 0.80. The Turkish validation of the scale was made by the authors of the article. In this context, the method suggested by Brislin (1970) was used. Experts in the field made Turkish-English and English-Turkish translations of the expressions. Following the translation, the scale was applied to the pilot and the original sample. The construct validity of the scale was determined using exploratory factor analysis. The scale’s KMO value was 0.850 as a result of the study, and the Barlett test was significant (*χ*^2^ = 1088.811; *p* < 0.000), indicating that the data were eligible for factor analysis. With factor loads ranging from 0.869 to 0.915, the exploratory factor analysis yielded a single factor structure that explained 80.174% of the overall variance. The results were found to be coherent with the single factor structure of the scale after confirmatory factor analysis. The results of confirmatory factor analysis are given in the findings section.

### Innovative behavior scale

The participants’ innovative behavior was measured by using the Innovative Behavior Scale, developed by Scott and Bruce (1994) and converted into Turkish by Akkoç (2012). The scale is a 5-point Likert type with one dimension and six items (1 = strongly disagree, 5 = strongly agree). Some of the expressions of the scale are as follows: “I come up with innovative concepts.” “I seek and allocate resources for new ideas.” In the Turkish version, Cronbach’s alpha reliability coefficient was calculated to be 0.82.

### The scale of supervisor support

The Supervisor Support Scale developed by Giray and Şahin (2012) was used to measure supervisor support. The scale is a 5-point Likert type with one dimension and 11 items (1 = Strongly disagree, 5 = Strongly agree). Some of the expressions of the scale are as follows: “When I make an unintentional error, my supervisor supports me against others in the organisation.” “My supervisor takes my opinions into account.” The scale’s Cronbach’s alpha reliability coefficient was determined to be 0.940.

## Results and discussion

**Figure 1:**
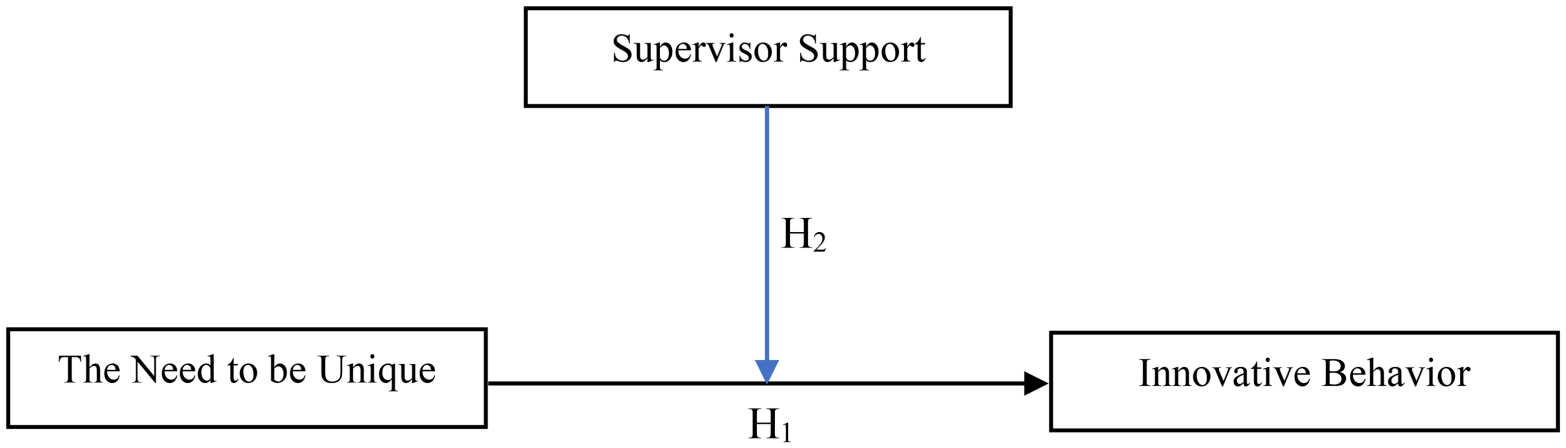
Research model.

CFA was performed to determine whether the observed variables represent latent variables (Hair et al., 2010). ∆*χ*^2^/sd, RMSEA, CFI and GFI indexes were used to determine whether the model structure was suitable for the data. The one-dimensional model’s goodness-of-fit values for the need to be unique scale ∆*χ*^2^/sd = 3.409, RMSEA = 0.080, CFI = 0.996, GFI = 0.991; values of the one-dimensional model’s goodness-of-fit for the inventive behavior scale ∆*χ*^2^/sd = 2.357, RMSEA = 0.060, CFI = 0.992, GFI = 0.983; for the one-dimensional manager support scale, it was calculated as ∆*χ*^2^/sd = 3.432, RMSEA = 0.080, CFI = 0.981, GFI = 0.937. These figures prove that the scales chosen are suitable and compatible with the data (Figure 1).

Because the data had a normal distribution, Maximum Likelihood Estimation (MLE) was utilized. According to the CFA outputs, the factor loads of the need to be different scale ranged from 0.809 to 0.896, the factor loads of the innovative behavior scale were between 0.745 and 0.841, and the factor loads of the supervisor support scale ranged from 0.820 to 0.921, all of which were statistically significant. The fact that these loads are greater than 0.5 indicates that convergent validity is provided (Bentler and Bonett, 1980; Bollen, 1989; Hair et al., 2010; Abubakar et al., 2017).

Convergent and discriminant validity were examined after the CFA analysis to determine to construct validity. If the mean Average Variance Extracted (AVE) are above 0.50, and the Composite Reliability (CR) values are equal to or greater than 0.60, this indicates convergent validity (Hair et al., 2014, p. 619; Çiçek and Işık, 2019, p. 2817); the fact that the square root of the AVE is greater than the correlation between the factors and the mean of the square of the Maximum Squared Variance (MSV) and the Average Shared Square Variance (ASV) is less than the AVE, shows discriminant validity (Fornell and Larcker, 1981; Anderson and Gerbing, 1988). Internal consistency (Cronbach’s alpha) values greater than 0.70 indicate a sufficient level of internal reliability. The mean and standard deviation values, correlation coefficients, reliability and discriminant validity of the scale are shown in Table 1. The square root of the AVE values is shown in diagonal brackets in Table 1. In Table 1, convergent and discriminant validity is provided, and when the correlation coefficients are examined, it is seen that there is a weak relationship between the need to be unique and innovative behavior, and supervisor support.

**Table 1:** Data with mean, standard deviation, correlation coefficients, confidence and discrimination validity.

Variable	Mean	SD	1	2	3
The need to be unique	2.972	1.000	**(0.857)**		
Innovative behavior	3.991	0.614	0.281**	**(0.785)**	
Supervisor support	3.632	0.909	0.006	0.166**	**(0.880)**
Cronbach’s alpha coefficient of confidence			0.917	0.907	0.974
Composite reliability (CR)			0.917	0.906	0.974
Average variance extracted (AVE)			0.736	0.617	0.776
Maximum squared variance (MSV)			0.078	0.078	0.027
Average shared square variance (ASV)			0.039	0.053	0.013

^*^p < 0.05, ^**^p < 0.01. The diagonal values in (bold) represent that discriminant validity is established.

Regression analysis was performed to determine the effect of the independent variable on the dependent variable. The results of the regression analysis were found to be statistically significant (*F*_(1,378)_ = 32.488, *p* < 0.000), and the need to be different had a significant and positive (*β* = 0.281, *p* < 0.000) effect on innovative behavior. The *R*^2^ value was calculated as 0.079. As a result, 8% of the diversity in inventive behavior can be attributed to the desire to stand out. H_1_ has been accepted.

The moderating influence of management support on the effect of the need to be unique on innovative behavior was investigated using hierarchical regression analysis. In order to test the hypotheses, firstly, the independent variable and the moderator variable were standardized, and the interactional variable was created using ±1 standard deviation (Aiken et al., 1991). In the first step of the regression analysis, which was carried out in three stages, the effect of the need to be unique on innovative behavior was investigated. The model is expanded in the second step to provide supervisor support. In the third stage of the regression analysis to determine the moderator effect, the interactional variable (Need to be unique*Supervisor support) suggested by Baron and Kenny (1986) was included in the model. It was observed that the interactional variable did not significantly affect innovative behavior (*β* = −0.014, *p* > 0.05). As a result, supervisor support does not appear to have a moderating influence on the effect of the drive to stand out on innovative behavior. H_2_ is not accepted. Table 2 shows the results that were achieved.

**Table 2:** Regression results.

	Innovative behavior		
	Model 1	Model 2	Model 3
The need to be unique	0.173***	0.172***	0.175***
Supervisor support		0.101**	0.102**
The need to be unique * Supervisor support			−0.014
*R* ^2^	0.077	0.101	0.099
*F*	32.488***	22.356***	14.955***

^***^p < 0.01, ^**^p < 0.05.

People are social beings that attempt to belong to a group, but they also try to be different from the crowd and feel unique. Innovative behavior should also be considered among the issues that will make people feel special and differentiate them from others in the workplace. Because new ideas and new practices are possible by behaving outside of routine practices and rules, it can be argued that such behaviors are made by people who want to differentiate themselves from others. Allowing such behaviors is only possible with the support of the organization and the supervisor. Considering that innovative products and services are essential to provide a competitive advantage, supervisors must support employees in displaying innovative behavior. In this context, creating an environment where employees can exhibit innovative behavior is necessary.

This study determined that the need to be unique has a positive and significant effect on innovative behavior. This is a predicted result for innovative behavior associated with innovation and creativity. This finding is relevant to advancing previous studies (e.g., Burns and Brady, 1992; Burns and Krampf, 2015). It has been evaluated that one of the factors that increase or decrease the severity of the relationship between the need to be unique and innovative behavior is the supervisor’s support. As stated in the literature, supervisor support is among the essential elements that reveal creativity (e.g., Scott and Bruce, 1994; Carnevale et al., 2017). And, it has been determined that employees need authority to exhibit innovative behavior (e.g., Sazkaya and Dede, 2018). However, in this study, it was observed that the supervisor support did not moderating role between the need to be unique and the innovative behavior. This result somewhat surprising for us. Nonetheless, our finding may not be interpreted as evidence that disregards the importance of supervisor support. This may be since the study was conducted in the education sector. These findings may have been influenced by factors including the teachers’ application of educational grade-appropriate lesson plans, the supervision’s lack of influence as long as the curriculum was followed, and the standardization of the course materials. It has been evaluated those activities were neither supported nor prevented by the supervision of people who need to plan course hours, improve the educational environment and conditions, and have different administrative duties for employees’ rights, to feel unique and different.

## Conclusion

This study examined the effect of the need to be unique on innovative behavior and the moderating role of supervisory support in this effect. In this context, applied research was conducted on Mersin’s employees in the education sector. As a result of the research, it has been determined that the need to be unique has a positive and considerable impact on innovative behavior; however, supervisor support does not have moderator role on the relationship between the need to be unique and the innovative behavior.

### Research implication

The need to be different leads to innovative behavior. But in collectivist cultures, it is not accepted easily that people are different from others. Conflicts can emerge or develop when teachers are involved in innovation. On the other hand, it is not possible to be successful without innovation. In addition, people with a high need for uniqueness can always be expected to exhibit innovative behavior. For this reason, supervisor should not hinder teachers’ innovative behavior. Therefore, teachers who exhibit innovative behavior should be supported. This support must first be provided by the supervisor. If the supervisor supports innovative behavior, it will be easier for others to accept and exhibit innovative behavior. On the other hand, the innovative behavior of teachers does not depend on only one factor. Organizational factors such as culture, organizational climate, leadership, training, giving freedom to make decision, communication among all stakeholders, and regulation of feedback within the organization can create an environment that fosters innovative behavior. It is a fact that supervisors in the education sector have an important role in determining the quality of education. For this reason, it is very important that school supervisors support teachers’ innovative behavior and create an environment where teachers can exhibit innovative behavior. In this case, teachers will fulfill their need for uniqueness and will be more satisfied with their work.

### Recommendations for future research

This study might provide different findings if it were conducted on individuals who work in different sectors and at different levels of supervision. Knowing and supporting employees’ expectations about being unique and special can also increase employee motivation, job satisfaction and performance. In this context, it can be recommended to examine variables such as the effect of the need to be unique on variables such as motivation, job satisfaction, performance, organizational support and the support of colleagues, leadership styles and the role of culture in the future studies.

## Limitations

There are also certain limitations to this study. The research’s primary limitations are that it is not longitudinal, it was only conducted in the education sector, and it was limited to a certain geographic area. The restricted nature of the sample limits generalizability of the findings. For this reason, conducting the research in other sectors and evaluating the data obtained at different times may lead to different outcomes.

The exclusive reliance on self-reports and the same questionnaire might have led to self-serving and common method biases (Podsakoff et al., 2003). Although several statistical diagnostics supported that method bias cannot totally account for our findings, there is a need to replicate this study to ascertain the validity of its findings. The potential operation of a self-serving bias also can be identified and reduced by examining the correlations between relevant items and a social desirability scale (Tourangeau and Yan, 2007).

## Data availability statement

The raw data supporting the conclusions of this article will be made available by the authors, without undue reservation.

## Author contributions

All authors listed have made a substantial, direct, and intellectual contribution to the work and approved it for publication.

## Conflict of interest

The authors declare that the research was conducted in the absence of any commercial or financial relationships that could be construed as a potential conflict of interest.

## Publisher’s note

All claims expressed in this article are solely those of the authors and do not necessarily represent those of their affiliated organizations, or those of the publisher, the editors and the reviewers. Any product that may be evaluated in this article, or claim that may be made by its manufacturer, is not guaranteed or endorsed by the publisher.
